# Doctors’ characteristics and the use of long consultations at out-of-hours services 2008–2017: a registry-based follow-up study in Norway

**DOI:** 10.1080/02813432.2019.1639929

**Published:** 2019-07-11

**Authors:** Hogne Sandvik

**Affiliations:** National Centre for Emergency Primary Health Care, NORCE Research AS, Bergen, Norway

**Keywords:** Emergency care, general practice, Norway, out-of-hours medical care, physician’s practice pattern, time management

## Abstract

**Objective:** The aim was to analyse whether there was a change in percentage of long consultations over a 10-year period, and whether individual doctors changed their use of time as they got more experience and specialisation during the same period.

**Design and setting:** This is a registry based study encompassing all consultations in primary care out-of-hours service in Norway in 2008 and 2017.

**Subjects:** For both years all doctors were included in cross sectional analyses. In addition, doctors who participated both years were included in a separate follow-up analysis.

**Main outcome measures:** Long consultations (>20 min) were identified by a time fee in the claims’ database.

**Results:** There were 4610 doctors in 2008 and 5620 in 2017, 904 participated both years. In 2008 a time fee was claimed in 38% of consultations, in 2017 in 47%. Older doctors made less use of the time fee, as did doctors who had many consultations, regular general practitioners, and general practice specialists. The general practitioners who participated both years increased their use of the time fee from 33% to 38% of consultations. Those who specialised in general practice during the 10-year period increased their use of the time fee from 34% to 37%.

**Conclusions:** Experienced doctors have fewer long consultations than inexperienced doctors. Over years there is a strong trend towards increasing the use of time fee during out-of-hours consultations. This trend is only partly offset by increasing the experience of the doctors.

**KEY POINTS**
Although consultation length may be associated with patient satisfaction there is also a cost-efficiency aspect to be taken into account
•Percentage long consultations out-of-hours increased from 38% in 2008 to 47% in 2017
•Experienced doctors had fewer long consultations
•Experience only partly offset the trend towards more long consultations

## Introduction

Consultation length has been found to be associated with patient satisfaction [1,2]. It has also been suggested as a proxy measure of quality of care [3]. Traditionally, general practitioners (GPs) in United Kingdom (UK) used to have only 5–6 min for each consultation, including time for completing the records [4,5]. More recent studies indicate that average consultation length in the UK is now approximately 10 min, and the relationship between consultation length and patient experience is less evident [6].

Length of consultations varies widely between different countries [7]. In Sweden average consultation length has been reported to be more than 20 min [8,9]. In this setting no association has been found between consultation length and patient satisfaction.

Thus, it seems that the association between consultation length and satisfaction is only present to a certain length, beyond which there is no more gain. Furthermore, from a cost-efficiency aspect unnecessarily long consultations should be avoided. Considering the large number of consultations performed in general practice every year, surveillance of consultation length is important for containing cost.

When comparing the performance of individual physicians in their own practices, one has to address the problem of case mix [10]. Doctors who are more patient centred or more interested in psychosocial issues, will probably use more time, and may attract patients who find these aspects attractive. The patients will differ from doctor to doctor, and this may explain why practice patterns differ between doctors. Therefore, it is difficult to compare the clinical performance of GPs without case mix adjustment [2].

However, out-of-hours (OOH) emergency health care is organised in a way that patients cannot choose which doctor to consult, thus eliminating the problem of case mix. In this setting, it is possible to do more valid analyses of doctors' actual performance.

In a previous Norwegian study it was found that experienced general practitioners and general practice specialists spent less time per patient than other doctors in the out-of-hours service [11]. However, this was a cross-sectional study, and it does not necessarily mean that, over time, specialisation and experience will cause the individual GP to spend less time on each consultation.

The aim of the present study was to analyse whether there was a general trend in time spent on consultations over a 10-year period in Norwegian OOH service, and whether individual GPs changed their use of time as they got more experience and specialisation during the same 10-year period.

## Material and methods

### Organization of Norwegian health care

Regular general practitioners (RGPs) act as gate keepers, and are organized in a list system, encompassing more than 99% of the inhabitants. Some municipalities have their own OOH service, while others cooperate. RGPs are obliged to do OOH work, but other physicians may also participate.

After every consultation the doctor sends an electronic compensation claim to the Norwegian Health Economics Administration (HELFO). Electronic billing has been compulsory since 2006. Thus, the present material encompasses all OOH consultations during the study period. Compensation claims include a time fee when the consultation lasts more than 20 min. In 2017 this fee was 174 NOK (approximately 20 USD). In 2008 the fee was 130 NOK.

### Study design

The National Centre for Emergency Primary Health Care receives annual OOH-data from HELFO in order to produce annual statistics [12]. These data are anonymized, but since 2008 HELFO has included a pseudo-id based on the doctor’s ID-number. This enables us to follow doctors’ compensation claims through consecutive years. Municipalities are categorized into five groups based on the number of inhabitants, and by centrality, which is a municipality’s geographical location in relation to a centre with important central functions, where 0 is least central (rural) and 3 is most central (urban) [13].

The present study was based on data used for the annual statistics from 2008 and 2017. The number of consultations remained stable throughout the period, with 1,323,453 consultations in 2008 and 1,332,024 in 2017.

### Variables

For each doctor the number of OOH consultations during a year was calculated, and the percentage of the consultations that resulted in a time fee, i.e. consultations that lasted more than 20 min. For explanatory variables the doctors were grouped by age (<35 years, 36–49 years, and ≥50 years), gender, RGP or other type of doctor, GP specialist or not, centrality, number of inhabitants in municipality, and the number of OOH consultations the doctor had during the year (<60, 60–149, 150–299, >299).

### Statistical analysis

In addition to frequency analyses, multiple logistic regression analyses were performed using the same explanatory variables. All variables were included in the model. The dependent variable was higher vs. lower than the median percentage use of the time fee (i.e. ‘slow’ versus ‘fast’ doctors).

These analyses were performed for all OOH doctors in 2008 and 2017 (Table 1) and for only doctors who did OOH consultations both in 2008 and 2017 (Table 2). In addition, I analysed the change in time fee percentage for individual doctors who did OOH consultations both in 2008 and 2017 (Table 3).

**Table 1.** Percentage long consultations (>20 min) in out-of-hours service during 2008 and 2017. Dependent variable in logistic regression: higher vs. lower percentage than the median value (0.34 in 2008 and 0.46 in 2017). All doctors are included both years.

	2008					2017				
				Logistic regression					Logistic regression	
Explanatory variables	*N*	Average number of consultations per doctor	Percentage of consultations where time fee was used	OR	95% CI	*N*	Average number of consultations per doctor	Percentage of consultations where time fee was used	OR	95% CI
Age groups										
≤35 years	2125	256	0.41	Ref.		2642	225	0.51	Ref.	
36–49 years	1547	314	0.37	0.96	0.83–1.12	2138	254	0.45	0.97	0.84–1.11
≥50 years	938	279	0.31	0.67	0.55–0.82	840	292	0.41	0.63	0.52–0.76
Gender										
Male	3080	330	0.37	Ref.		3052	314	0.46	Ref.	
Female	1530	180	0.39	1.00	0.87–1.14	2568	166	0.49	1.05	0.94–1.18
Type of doctor										
Other doctor	1907	278	0.42	Ref.		1962	272	0.51	Ref.	
Regular general practitioner	2703	282	0.35	0.79	0.69–0.91	3658	233	0.45	0.78	0.68–0.88
GP specialist										
No	3340	277	0.41	Ref.		4270	245	0.50	Ref.	
Yes	1270	287	0.29	0.51	0.43–0.60	1350	251	0.37	0.47	0.40–0.55
Centrality^a^										
0 Rural	1304	155	0.48	Ref.		1286	145	0.57	Ref.	
1	465	204	0.38	0.88	0.68–1.13	601	182	0.47	0.76	0.60–0.97
2	1018	299	0.31	0.53	0.42–0.67	1341	259	0.41	0.45	0.36–0.57
3 Urban	1823	379	0.34	0.63	0.50–0.80	2392	311	0.46	0.55	0.43–0.70
Number of inhabitants										
<2001	344	114	0.50	Ref.		248	100	0.66	Ref.	
2001–5000	780	169	0.47	1.11	0.83–1.49	652	155	0.58	0.77	0.53–1.11
5001–10,000	740	193	0.43	1.05	0.78–1.43	763	162	0.50	0.43	0.30–0.61
10,001–50,000	1684	328	0.30	0.45	0.33–0.63	2480	261	0.41	0.33	0.23–0.49
>50,000	1062	400	0.35	0.59	0.41–0.83	1477	331	0.48	0.47	0.32–0.71
Number of consultations										
<60	1152	28	0.43	Ref.		0.51	27		Ref.	
60–149	1176	101	0.39	0.93	0.78–1.11	0.48	99		0.86	0.74–1.00
150–299	1040	214	0.36	0.82	0.68–0.99	0.44	209		0.64	0.55–0.75
>299	1237	744	0.33	0.72	0.60–0.87	0.42	790		0.57	0.49–0.67
Total	4610	280	0.38			5620	246	0.47		

^a^Centrality is a municipality’s geographical location in relation to a centre with important central functions, where 0 is least central (rural) and 3 is most central (urban).

**Table 2.** Percentage long consultations (>20 min) in out-of-hours service during 2008 and 2017. Dependent variable in logistic regression: higher vs. lower percentage than the median value (0.30 in 2008 and 0.34 in 2017). Only doctors who participated both years are included.

	2008					2017				
				Logistic regression					Logistic regression	
Explanatory variables	*N*	Average number of consultations per doctor	Percentage of consultations where time fee was used	OR	95% CI	*N*	Average number of consultations per doctor	Percentage of consultations where time fee was used	OR	95% CI
Age groups										
≤35 years	312	365	0.35	Ref.		0	–	–	–	–
36–49 years	466	374	0.33	0.87	0.61–1.22	533	221	0.39	Ref.	
≥50 years	126	551	0.29	0.53	0.31–0.89	371	294	0.36	0.67	0.50–0.90
Gender										
Male	647	470	0.32	Ref.		647	294	0.37	Ref.	
Female	257	208	0.35	0.99	0.71–1.37	257	142	0.40	1.13	0.82–1.55
Type of doctor										
Other doctor	0	–	–	–	–	0	–	–	–	–
Regular general practitioner	904	396	0.33	–	–	904	251	0.38	–	–
GP specialist										
No	637	384	0.37	Ref.		209	230	0.46	Ref.	
Yes	367	413	0.27	0.58	0.41–0.81	695	257	0.35	0.42	0.30–0.60
Centrality^a^										
0 Rural	197	226	0.46	Ref.		177	187	0.49	Ref.	
1	94	251	0.34	0.51	0.28–0.94	94	212	0.37	0.73	0.39–1.38
2	246	399	0.28	0.27	0.16–0.46	266	255	0.33	0.44	0.25–0.78
3 Urban	367	522	0.29	0.41	0.24–0.71	367	289	0.36	0.57	0.31–1.05
Number of inhabitants										
<2001	32	252	0.46	Ref.		22	87	0.55	Ref.	
2001–5000	117	255	0.46	1.23	0.47–3.20	101	187	0.51	0.76	0.20–2.91
5001–10,000	157	239	0.40	1.09	0.42–2.78	135	184	0.43	0.38	0.10–1.42
10,001–50,000	371	397	0.28	0.58	0.22–1.54	420	277	0.33	0.24	0.06–0.92
>50,000	227	595	0.27	0.41	0.15–1.14	226	286	0.36	0.28	0.07–1.10
Number of consultations										
<60	119	30	0.39	Ref.		277	25	0.43	Ref.	
60–149	195	106	0.34	0.79	0.48–1.31	222	101	0.39	1.31	0.89–1.91
150–299	238	216	0.34	0.71	0.44–1.16	192	209	0.37	0.96	0.64–1.43
>299	349	806	0.29	0.71	0.45–1.14	213	739	0.30	0.52	0.35–0.77
Total	904	396	0.33			904	251	0.38		

^a^Centrality is a municipality’s geographical location in relation to a centre with important central functions, where 0 is least central (rural) and 3 is most central (urban).

**Table 3.** Change in percentage long consultations (>20 min) for individual doctors who participated in out-of-hours service both in 2008 and 2017. Dependent variable in logistic regression: higher vs. lower percentage than the median value (0.04).

			Logistic regression	
Explanatory variables	*N*	Difference in percentage time fee	OR	95% CI
Age groups 2008				
≤35 years	312	0.03	Ref.	
36–49 years	466	0.06	1.18	0.85–1.64
≥50 years	126	0.04	1.18	0.73–1.91
Gender				
Male	647	0.05	Ref.	
Female	257	0.05	1.23	0.90–1.68
Type of doctor 2008				
Other doctor	0	–	–	–
Regular general practitioner	904	0.05	–	–
GP specialist 2008				
No	637	0.04	Ref.	
Yes	367	0.06	1.20	0.88–1.65
Centrality 2008^a^				
0 Rural	197	0.01	Ref.	
1	94	0.02	0.88	0.50–1.55
2	246	0.05	1.37	0.84–2.23
3 Urban	367	0.07	1.44	0.86–2.41
Number of inhabitants 2008				
<2001	32	0.02	Ref.	
2001–5000	117	−0.01	0.84	0.36–1.94
5001–10,000	157	0.04	1.54	0.68–3.51
10,001–50,000	371	0.05	1.38	0.58–3.29
>50,000	227	0.09	1.57	0.64–3.90
Number of consultations 2008				
<60	119	0.04	Ref.	
60–149	195	0.07	1.41	0.88–2.25
150–299	238	0.04	1.07	0.68–1.69
>299	349	0.05	0.94	0.60–1.47
Total	904	0.05		

^a^Centrality is a municipality’s geographical location in relation to a centre with important central functions, where 0 is least central (rural) and 3 is most central (urban).

This material is complete, and the differences identified are real and not fraught with statistical uncertainty. The data are therefore presented without confidence intervals and no statistical tests have been undertaken, except for the logistic regression analyses where odds ratios are presented with 95% confidence intervals (95% CI).

## Results

There were 4610 OOH doctors in 2008, of whom 2 703 (59%) were RGPs. In 2017 there were 5620 OOH doctors, of whom 3 658 (65%) were RGPs. There were 904 doctors who participated in the OOH services both in 2008 and 2017. All of them were RGPs. In 2008 only 57 doctors were ≥65 years (1.2%), the corresponding number in 2017 was 107 (1.9%).

In 2008 OOH doctors had on average 280 consultations, and claimed a time fee in 38% of the consultations (Table 1). The corresponding numbers in 2017 were 246 and 47%. Comparison of different groups of doctors resulted in the same pattern in 2008 and 2017: Female doctors had significantly fewer consultations than their male colleagues and there was a slight tendency that female doctors used the time fee more often. However, this was not significant when correcting for other variables in the multiple regression analysis. Older doctors made less use of the time fee, as did RGPs and GP specialists. Doctors ≥65 years did not differ significantly from those ≥50 years. OOH doctors who had many consultations used the time fee less often than those who had fewer consultations. Time fee was more often used in smaller and rural areas.

Table 2 shows corresponding results only for the 904 doctors who did OOH service both in 2008 and 2017, all of them RGPs. In 2008 they had on average 396 consultations, and claimed a time fee in 33% of the consultations. The corresponding numbers in 2017 were 251 and 38%. Comparison of different groups of doctors resulted in the same pattern as for all OOH doctors.

On average the 904 RGPs who did OOH service both in 2008 and 2017 increased their use of the time fee by five percentage points (Table 3). There was a tendency that the increased use of time fee was largest in the most urban and central areas. However, these differences were not statistically significant in the logistic regression analysis. The 337 RGPs who were not certified GP specialist in 2008, but gained this speciality during the period, increased their use of time fee from 34% to 37%.

## Discussion

The cross sectional analyses, both in 2008 and 2017, showed the same results: Experienced doctors used less time in consultations than inexperienced doctors. Indicators of experience are age, number of consultations, and specialisation in general practice. Being a RGP also indicates experience, since work in regular general practice closely resembles OOH work.

However, there was a considerable change in the use of time fee from 2008 to 2017, an increase from 38% to 47% of all consultations. We have no indications that the average doctor was more experienced in 2008 than in 2017. Even those doctors (all RGPs) who participated in OOH services both years, and therefore had gained a lot of experience, increased their use of the time fee from 33% to 38%. Those who specialized in general practice during the period increased their use of the time fee from 34% to 37%.

### Strengths and limitations

The main strength of this study is that it encompasses all OOH doctors in Norway, both in 2008 and 2017, and that it is possible to follow the practice pattern of individual doctors over time. Furthermore, the case mix problem, usually encountered when analysing the practice pattern of individual GPs in their own practices, is avoided [2,10].

The number of explanatory variables used in the multiple logistic regression analyses could pose a problem of over controlling. However, the relatively stable results in two independent samples (2008 and 2017) indicate that this is not a real limitation.

An important limitation is that the length of consultations is not recorded exactly (number of min), but only as consultations lasting more or less than 20 min. As consultations lasting more than 20 min are honoured by a time fee, it cannot be ruled out that doctors may be tempted to use it more than they are entitled to. However, this can hardly explain the difference between groups or change over time.

Since average length of consultations varies between different countries [7], the results presented here cannot directly be generalized to other countries. However, the trend towards longer consultations has also been demonstrated in other countries [6], and the expansion of defensive medicine is a general problem [14–18].

### Findings in relation to other studies

Similar findings regarding doctors’ use of time in relation to their experience and age have been reported from Norwegian OOH services [11,19]. A study from Australian general practice found that GPs ≥65 years had longer consultations, as did female GPs and GPs working in small, rural practices [20]. In a study of six European countries it was found that 22% of the variance in consultation length could be ascribed to doctor variables [7]. In the same study the GPs’ age or gender did not matter significantly, but consultations lasted longer in city practices than in rural practices. Furthermore, increased doctors’ workload was associated with shorter consultations. A review of 33 studies, mostly from the US, indicated that female physicians tend to have longer consultations than male physicians [21]. UK studies indicate that female GPs and GPs ≥65 years have longer consultations than male or younger GPs [22,23]. A negative correlation has been found between length of consultations and the GPs’ length of registration with the General Medical Council [22], probably reflecting the GPs’ experience.

When patients choose which doctor to consult, different case mix may explain the conflicting findings. Female doctors may have a more patient-centred approach and therefore may attract patients who need more time during consultations [21].

There has been a trend towards increasing the consultation time also in the UK [6], but this has been on a different level than what is happening in Norwegian OOH services. Many problems presented OOH (e.g. respiratory or urinary tract infections) are easily handled and it is surprising that the average OOH doctor needs more than 20 min for almost half of the consultations.

Although the frequency of OOH patients with acute otitis media has been somewhat reduced, urinary and respiratory tract infections are just as frequent in 2017 as in 2008. The number of psychiatric patients, who often are time consuming, also remains stable. Thus, there are no indications that the problems presented OOH are more complex in 2017 than in 2008 [12].

### Possible implication

The most likely explanation for this trend is defensive medicine and overtreatment. OOH work is often performed under difficult circumstances, with an increased risk of making errors [24]. Increasing medicolegal concerns and fear of complaints may cause doctors to behave more defensively in their practice [14–18]. Doctors order more tests, more imaging technology, refer more patients, prescribe more unnecessary drugs, and make more detailed notes for documenting that no errors have been committed.

## Conclusion

There is a strong trend towards increasing the use of the time fee during OOH consultations. This trend is only partly offset by increasing the experience of the doctors. Further studies should investigate the role medicolegal concerns play in this costly development.
